# Pre-Discharge Predictors of 1-Year Rehospitalization in Adolescents and Young Adults with Severe Mental Disorders: A Retrospective Cohort Study

**DOI:** 10.3390/medicina56110613

**Published:** 2020-11-15

**Authors:** Francesco Bartoli, Daniele Cavaleri, Federico Moretti, Bianca Bachi, Angela Calabrese, Tommaso Callovini, Riccardo M. Cioni, Ilaria Riboldi, Renata Nacinovich, Cristina Crocamo, Giuseppe Carrà

**Affiliations:** 1 Department of Medicine and Surgery, University of Milano Bicocca, via Cadore 48, 20900 Monza, Italy; d.cavaleri1@campus.unimib.it (D.C.); f.moretti24@campus.unimib.it (F.M.); b.bachi@campus.unimib.it (B.B.); a.calabrese16@campus.unimib.it (A.C.); t.callovini@campus.unimib.it (T.C.); r.cioni1@campus.unimib.it (R.M.C.); i.riboldi1@campus.unimib.it (I.R.); renata.nacinovich@unimib.it (R.N.); cristina.crocamo@unimib.it (C.C.); giuseppe.carra@unimib.it (G.C.); 2 Division of Psychiatry, University College London, Maple House 149, London W1T 7BN, UK

**Keywords:** rehospitalization, adolescents, young adults, severe mental disorders, substances, suicide

## Abstract

*Background and objectives*: Readmissions of youths hospitalized for a severe mental disorder are common events and bear a remarkable human, social, and economic burden. The current study aimed at evaluating predictors of 1-year rehospitalization in a sample of adolescents and young adults with severe mental disorders. *Materials and Methods*: Data for ≤25-year-old inpatients with a severe mental disorder and consecutively admitted between 1 January 2016 and 30 June 2019 were collected. Subjects were retrospectively assessed over a follow-up period of one year after the index discharge to track readmissions—i.e., the primary outcome variable. Standard descriptive statistics were used. The association between variables and 1-year rehospitalization was estimated using the univariate Cox proportional hazards regression model. We then carried out a multivariable Cox regression model, also estimating the covariate-adjusted survivor function. Hazard ratios (HRs) with related 95% confidence intervals (95% CIs) were provided. *Results*: The final sample included 125 individuals. The multivariable Cox regression model estimated that co-occurring substance use disorders (HR = 2.14; 95% CI: 1.08 to 4.26; *p* = 0.029) and being admitted for a suicide attempt (HR = 2.49; 95% CI: 1.13 to 5.49; *p* = 0.024) were both significant predictors of 1-year rehospitalization. *Conclusions*: Our study showed that comorbid substance use disorders and being admitted for a suicide attempt were predictors of early readmission in youths with severe mental disorders. Although their generalizability is limited, our findings could contribute to improve the quality of young patients’ mental health care by identifying vulnerable subjects who may benefit from tailored interventions to prevent rehospitalizations.

## 1. Introduction

A substantial number of inpatients hospitalized for a severe mental disorder is readmitted early after discharge [1,2]. Because of their human, social, and economic burden, rehospitalizations are generally seen as a negative outcome, implying that the original admission somehow failed to adequately treat the patient [3]. Therefore, readmission rates are considered a proxy indicator of the adequacy of mental health care [1,3]. This turns out to be particularly relevant for young individuals’ care, as most severe mental disorders originate in adolescence and early adulthood [4]. Recurrent hospitalizations of young patients are disruptive to families, interfere with school and work activities, and are emotionally and physically distressing [5]. Recent studies estimate that more than a third of youths will be readmitted to a psychiatric ward within one year after discharge [6,7]. Thus, it seems crucial to assess factors that are likely to influence readmission rates [5], with a specific focus on important factors available from routine clinical practice.

Research has explored a wide range of variables that could predict future readmissions to inpatient mental health services among children and adolescents, taking into consideration both socio-demographic and clinical characteristics [8,9]. However, the available evidence is extremely heterogeneous in terms of study design, target population, data source, and combination of considered characteristics [6,10,11]. Previous studies showed mixed findings, making it difficult to draw definitive conclusions about these potential predictors [9,12]. Furthermore, most of the studies are focused on children and adolescents [9], with few data from individuals in their late adolescence and early twenties. Research in this field is important to define individual predictors of early readmissions that may be useful to personalize interventions.

Specifically focusing on this often overlooked age group, our study aimed at evaluating a broad set of factors potentially associated with 1-year rehospitalization in a sample of adolescents and young adults with severe mental disorders consecutively discharged from two general inpatient services of a large Mental Health and Addiction Department in Milan metropolitan area, Italy. Our analysis investigated pre-discharge individual factors, including standard demographic (age and gender) and clinical characteristics (diagnosis, comorbid alcohol or substance use disorders, hospitalization due to a suicide attempt, previous contacts with mental health services, past psychiatric voluntary and compulsory hospitalizations, length of hospital stay, pharmacological medications prescribed at the end of inpatient care, and type of discharge planning and referrals), in order to assess their role as predictors of readmission. This would allow providing helpful information to prevent readmission occurrence and enhance the quality of young patients’ psychiatric care.

## 2. Materials and Methods

### 2.1. Study Design and Setting

This exploratory retrospective cohort study was drawn up following the STrengthening the Reporting of OBservational studies in Epidemiology (STROBE) checklist [13]. We included individuals consecutively admitted to two distinct inpatient mental health services of the local ASST Nord Milano Trust (accounting for a total of 27 beds, including one for <18 years old subjects), which provides treatment for approximately 270,000 inhabitants of the northern area of the Metropolitan City of Milan. The current study, as a part of the broader Northern Milan Area Cohort (NOMIAC) project, was notified to the local Ethics Committee.

### 2.2. Data Collection

Data were retrospectively collected between March and June 2020, using the electronic medical records of all individuals with a severe mental disorder who were discharged from the inpatient mental health services between 1 January 2016 and 30 June 2019 and were ≤25 years old at the time of admission. Available structured information on variables potentially associated with rehospitalization was collected. Patients were followed-up for one year after the discharge to track readmissions to inpatient mental health services. Anonymized data were used to fill in a standardized extraction template and double-checked for accuracy.

### 2.3. Inclusion Criteria

We included individuals who met the following inclusion criteria at the time of the index hospital admission: an age ≤25 years; a diagnosis of a severe mental disorder [14,15], namely schizophrenia spectrum or other psychotic disorders, major depressive disorder, bipolar disorder, or personality disorders, as defined by DSM-5 [16]. We excluded individuals who did not live in the Trust catchment area both at the first hospitalization and during the follow-up period, as well as those whose main data were incomplete, unclear or missing from the user’s record.

### 2.4. Outcome and Predictor Variables

The study outcome was the readmission (failure event) to inpatient mental health services within 365 days from the discharge date. Time at risk was set up in days from discharge to readmission over 1-year follow-up. Consistently with the literature [1], we defined “pre-discharge predictors” as any variable describing individual clinical characteristics before or during the index admission up to the discharge. According to the information available from clinical records, we considered the following key characteristics: age, gender, diagnosis, comorbid lifetime alcohol or substance use disorders, being admitted for a suicide attempt, previous contacts with mental health services, previous hospitalizations, current compulsory hospitalization, length of hospital stay, psychopharmacological prescription at discharge, considering main drug categories (oral or long-acting injectable antipsychotics (LAIs), mood stabilizers, antidepressants), and type of discharge planning and referrals. Selected ICD-10 diagnoses of severe mental disorders (F20 to F29; F30 to F39; F60 to F69) and co-occurring alcohol/substance use disorders (F10 to F19) were converted into the related DSM-5 mental disorder [16]. With regard to suicide attempts leading to the index admission, we considered only those involving self-injurious behaviors in which there was at least some intent to die [17]. Due to the lack of specific and reliable information from the individual clinical records, suicidal ideation and plans without any action taken, as well as nonsuicidal self-injury, were not considered.

### 2.5. Data Analysis 

Standard descriptive statistics, including proportion (%), mean (standard deviation (SD)) or median (interquartile range (IQR)), were used. The association between candidate variables and 1-year readmission was estimated using the univariate Cox proportional hazards regression model, after checking relevant assumptions. Then, we carried out a multivariable Cox regression model, estimating the covariate-adjusted survivor function. The covariate-adjusted function focuses on the survival considering reference category values of candidate variables. We retained candidate variables that showed a *p*-value < 0.1 at the univariate level. A Hazard ratio (HR) with related 95% confidence interval (95% CI) was provided for any selected variable. In order to limit a possible overfitting of the model, we followed the “one-in-ten rule”, including in the final model no more than one factor for every ten events [18]. The significance level was set at *p* < 0.05. Stata statistical software package release 16 [19] was used to perform the analyses.

## 3. Results

### 3.1. Study Participants and Sample Characteristics

A total of 156 individuals ≤25 years of age was admitted during the study period. Of them, 31 were excluded either because the medical record was not fully available (*n* = 4) or because they were living outside the Trust catchment area at the time of the index episode and/or during the 1-year follow-up (*n* = 27). The final sample included 125 individuals (mean age ± SD: 20.6 ± 2.9 years; proportion of men: 53.6%) suffering from a schizophrenia spectrum disorder (35.2%), major depressive disorder (10.4%), bipolar disorder (6.4%), or a personality disorder (48.0%). In addition, 17 (13.6%) and 45 (36.0%) had a comorbid alcohol or substance use disorder, respectively. Twenty subjects (16.0%) were admitted subsequently due to a suicide attempt. The majority of participants (68.8%) had already had contacts with mental health services and about a quarter (24.0%) had been hospitalized in a psychiatric setting. Twenty-six (20.8%) were compulsorily admitted. The median length of hospital stay was 11 days (IQR: 8–17 days). A total of 91 (72.8%) were prescribed antipsychotics at discharge, of which 74 (59.2% of the sample) received oral antipsychotics and 17 (13.6% of the sample) received LAIs, 27 (21.6%) had mood stabilizers, and 32 (25.6%) received any antidepressants. More than a quarter of the subjects (28.8%) reported at least one readmission during the 1-year follow-up period. The vast majority of the individuals were discharged at home (89.6%). The sample characteristics are reported in Table 1.

### 3.2. Univariate and Multivariable Cox Regression Model

**Figure 1:**
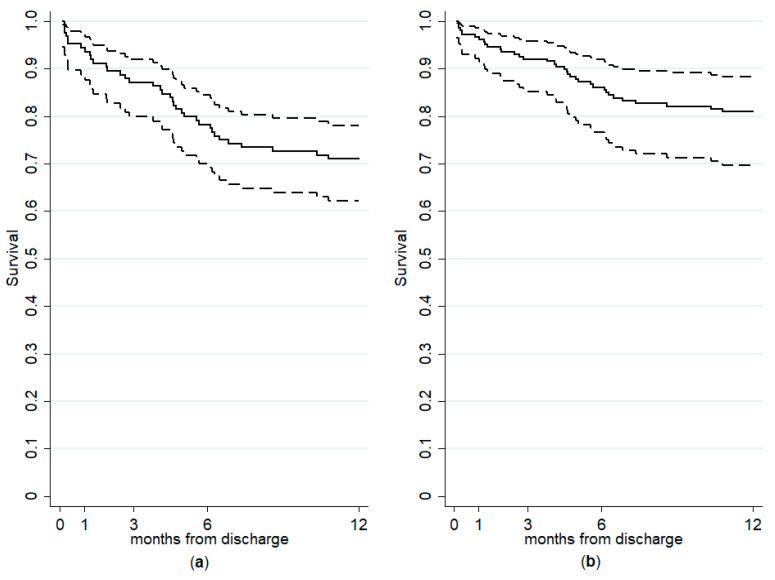
Multivariable Cox regression model: unadjusted (**a**) and covariate-adjusted * (**b**) survivor estimates with 95% confidence intervals. The difference between curves shows the joint effect of lifetime substance use disorder and admission due to a suicide attempts on 1-year readmission (failure event). * Adjusted for substance use disorders and admissions for a suicide attempt.

As a whole, 36 readmission events were recorded. At a univariate level, we found no differences between subjects readmitted within 1 year and those not readmitted in terms of age at study entry, diagnosis, suicide attempts, previous contacts with mental health services and hospitalizations, length of hospital stay, psychopharmacological treatment, and type of discharge planning and referrals. The demographic and clinical characteristics of the study sample, with related p-values from the univariate Cox proportional hazards regression models, are shown in Table 1. Two relevant clinical variables from univariate models, i.e., being admitted for a suicide attempt (*p* = 0.078) and having comorbid substance use disorders (*p* = 0.081), were included in the final model. The multivariable Cox regression model is reported in Table 2. We estimated that suffering from a lifetime substance use disorder (HR = 2.14; 95% CI: 1.08 to 4.26; *p* = 0.024) and being hospitalized due to a suicide attempt (HR = 2.49; 95% CI: 1.13 to 5.49; *p* = 0.029) were both significant predictors of 1-year readmission. The unadjusted and covariate-adjusted survivor functions after Cox regression are shown in Figure 1.

## 4. Discussion

### 4.1. Summary and Interpretation of Findings

This retrospective cohort study examined pre-discharge factors that may predict a readmission within one year from the index discharge among adolescents and young adults with severe mental disorders. Understanding the rates and predictors of rehospitalization is useful for health professionals in order to detect high-risk populations commendable of tailored interventions [1]. In our sample, about a third of the youths admitted to inpatient care was readmitted within the follow-up period of one year from the discharge. The readmission rates were sufficiently consistent with those reported among young adults in a recent analysis at a national level [7]. We found that comorbid substance use disorders and admissions for a suicide attempt may significantly predict an early rehospitalization, with a two-fold increase in the readmission rates. The size of these effects should be considered moderate to large, following recommended cut-offs to assess the HR magnitude [20], and seems consistent with current evidence [1,9]. As regards alcohol and substance use disorders, our sample shows rates that are considerably higher than those reported in the general population both at a national and at a European level [21], consistently with epidemiological data showing high rates of comorbid addictive behaviors among subjects with severe mental disorders [22]. A longitudinal analysis recently estimated that having a history of cannabis use when admitted to an early intervention inpatient unit for psychosis was associated with a higher number of subsequent hospital readmissions [23]. Furthermore, higher rates of readmission in individuals who started using drugs in their youth, compared to people who started later in their life, were estimated [24]. Our findings confirm that comorbid substance use disorders may lead to symptomatic relapse, impair clinical and psychosocial adjustment, reduce medication adherence, and lower the response to treatment among subjects with severe mental disorders [25,26]. As for suicidality, our analysis estimated that individuals who were admitted because of a suicide attempt were more likely to be rehospitalized, consistently with previous studies showing an association between suicidal behavior and readmission in both adolescents [9,12] and adults with early psychosis [27]. Youths with recent suicide attempts may receive more intensive care; therefore, readmissions may also reflect the risk as perceived by mental health care staff [12]. Further, we could hypothesize a reciprocal link between suicide attempts and substance use disorders in influencing the readmission rates of youths with severe mental disorders. Indeed, recent epidemiological evidence highlighted a bidirectional association between suicidality and substance abuse among adolescents and young adults [28,29,30], as well as in individuals with severe mental disorders [31,32]. Therefore, it is likely that the increase in readmission rates among adolescents and young adults with substance use disorders and who have attempted suicide may be at least partially explained by the additional burden of these clinical features on the course of severe mental disorders [33,34].

### 4.2. Limitations

Our findings should be interpreted with caution considering some methodological limitations. First, due to the study design, we could not assess post-discharge factors data (e.g., continuity of care, follow-up interventions, and compliance to pharmacological treatment) that may have had an impact on the likelihood of rehospitalization. Since nonadherence is a major concern in the outpatient treatment of severe mental disorders [35,36,37], especially in the early phases of the disease [38], studies with different designs are needed. Moreover, the limited sample size did not allow us to analyze the pharmacological therapy in more detail—for example, by evaluating the WHO-defined daily dose [39]. Second, despite medical databases being generally considered a robust tool for research [40], we should take into account that information bias may have partially affected the findings of our study, since data derived from medical records are obviously not as reliable as those obtained from standardized assessments. This reflects the complexity of studies based on real-world clinical settings, in which several factors may affect their internal validity [35]. Third, it should be noted that the Italian mental health care delivery system is grounded in a community-based model [41]. Accordingly, our findings, relying on a specific geographical catchment area, might have been influenced by unexplored area-level factors [42] that may have impacted the rates of rehospitalization [2]. More comprehensive data from different geographical areas are needed to provide a nation-wide picture and identify the possible influence of diverse organizational issues at a community level. Finally, due to the limited sample size, we could not estimate whether specific patterns of substance use disorders—in terms of severity and involved substances (cannabis, cocaine, novel psychoactive substances, others)—may have influenced the rates of readmission. Likewise, we could not evaluate the possible effects of other suicidal features, such as suicide ideation or planning, that do not necessarily share the same clinical outcomes of suicide attempts [43].

## 5. Conclusions

Suicide attempts leading to hospitalization and comorbid substance use disorders lead to increased rates of readmission among adolescents and young adults with severe mental disorders. Notwithstanding the limited generalizability, our findings could help clinicians to better understand which pre-discharge characteristics are predictive of an early readmission and deliver personalized interventions. Clinical routine practice could benefit from the implementation of psychosocial interventions in both healthcare and community settings for substance misuse [44] and suicide prevention [45]. As they cross the transition boundary between adolescent and adult services, youths are at high risk of disengagement and discontinuity of care [46]. Individuals at risk may benefit from tailored preventive efforts and enhanced discharge plans which could lower the rates of rehospitalization, as psychiatric readmission rates are likely responsive to quality improvement efforts [47]. Further research, using both qualitative and quantitative methods, is needed to better identify predictors of readmission, in order to develop innovative preventive strategies and improve outcomes for this high-risk population.

## Figures and Tables

**Table 1:** Participant characteristics at study entry.

Characteristics	Total Sample (*n* = 125)	Readmitted Within 1 Year (*n* = 36)	Not Readmitted (*n* = 89)	*p*-Value *
**Age at study entry**, years				0.727
Mean (SD)	20.6 (2.9)	20.7 (3.3)	20.6 (2.7)	
Median (IQR)	20.3 (18.4 to 22.8)	20.8 (17.9 to 23.6)	20.3 (18.5 to 22.6)	
**Male gender**	67 (53.6%)	20 (55.6%)	47 (52.8%)	0.911
**Diagnosis**				0.684
Schizophrenia spectrum or other psychotic disorder	44 (35.2%)	15 (41.7%)	29 (32.6%)	
Bipolar disorder	8 (6.4%)	2 (5.6%)	6 (6.7%)	
Major depressive disorder	13 (10.4%)	2 (5.6%)	11 (12.4%)	
Personality disorder	60 (48.0%)	17 (47.2%)	43 (48.3%)	
**Alcohol use disorders**	17 (13.6%)	7 (19.4%)	10 (11.2%)	0.170
**Substance use disorders**	45 (36.0%)	17 (47.2%)	28 (31.5%)	0.081
**Admission for a suicide attempt**	20 (16.0%)	9 (25.0%)	11 (12.4%)	0.078
**Previous contacts with mental health services**	86 (68.8%)	28 (77.8%)	58 (65.2%)	0.201
**Previous hospitalizations**				0.130
None	95 (76.0%)	24 (66.7%)	71 (79.8%)	
At least one	30 (24.0%)	12 (33.3%)	18 (20.2%)	
**Compulsory hospitalization**	26 (20.8%)	6 (16.7%)	20 (22.5%)	0.422
**Length of hospital stay**, days				0.504
Mean (SD)	16.7 (21.4)	14.8 (9.7)	17.5 (24.6)	
Median (IQR)	11 (8–17)	12.5 (9–17)	10 (7–16)	
**Psychopharmacological treatment**				
Antipsychotics	91 (72.8%)	29 (80.6%)	62 (69.7%)	0.311
LAIs ^§^	17 (13.6%)	8 (22.2%)	9 (10.1%)	0.152 ^§^
Mood stabilizers	27 (21.6%)	8 (22.2%)	19 (21.3%)	0.920
Antidepressants	32 (25.6%)	10 (27.8%)	22 (24.7%)	0.687
**Type of discharge planning and referrals**				0.626
Home	112 (89.6%)	33 (91.7%)	79 (88.8%)	
Residential facilities	13 (10.6%)	3 (8.3%)	10 (11.2%)	

SD = standard deviation; IQR = interquartile range; LAIs = long-acting injectable antipsychotics. * Derived from the univariate Cox proportional hazards regression models; ^§^ vs. oral antipsychotics.

**Table 2:** Multivariable Cox regression model.

Characteristics	HR	95% CI	*p*-Value
**Admission for a suicide attempt**	2.49	1.13 to 5.49	0.024
**Substance use disorders**	2.14	1.08 to 4.26	0.029

HR = Hazard Ratio; 95% CI = Confidence Interval.

## References

[1] Donisi V., Tedeschi F., Wahlbeck K., Haaramo P., Amaddeo F. (2016). Pre-discharge factors predicting readmissions of psychiatric patients: A systematic review of the literature. BMC Psychiatry.

[2] Kalseth J., Lassemo E., Wahlbeck K., Haaramo P., Magnussen J. (2016). Psychiatric readmissions and their association with environmental and health system characteristics: A systematic review of the literature. BMC Psychiatry.

[3] Rumball-Smith J., Hider P. (2009). The validity of readmission rate as a marker of the quality of hospital care, and a recommendation for its definition. N. Z. Med. J..

[4] Kessler R.C., Angermeyer M., Anthony J.C., De Graaf R., Demyttenaere K., Gasquet I., De Girolamo G., Gluzman S., Gureje O., Haro J.M. (2007). Lifetime prevalence and age-of-onset distributions of mental disorders in the World Health Organization’s World Mental Health Survey Initiative. World Psychiatry.

[5] Nakamura M.M., Toomey S.L., Zaslavsky A.M., Berry J.G., Lorch S.A., Jha A.K., Bryant M.C., Geanacopoulos A.T., Loren S.S., Pain D. (2014). Measuring pediatric hospital readmission rates to drive quality improvement. Acad. Pediatr..

[6] Phillips M.S., Steelesmith D.L., Campo J.V., Pradhan T., Fontanella C.A. (2020). Factors Associated With Multiple Psychiatric Readmissions for Youth with Mood Disorders. J. Am. Acad. Child Adolesc. Psychiatry.

[7] Tedeschi F., Donisi V., Salazzari D., Cresswell-Smith J., Wahlbeck K., Amaddeo F. (2020). Clinical and organizational factors predicting readmission for mental health patients across Italy. Soc. Psychiatry Psychiatr. Epidemiol..

[8] Fontanella C.A. (2008). The influence of clinical, treatment, and healthcare system characteristics on psychiatric readmission of adolescents. Am. J. Orthopsychiatry.

[9] Madden A., Vajda J., Llamocca E.N., Campo J.V., Gorham T.J., Lin S., Fontanella C.A. (2020). Factors associated with psychiatric readmission of children and adolescents in the U.S.: A systematic review of the literature. Gen. Hosp. Psychiatry.

[10] Miller D.A.A., Ronis S.T., Slaunwhite A.K., Audas R., Richard J., Tilleczek K., Zhang M. (2020). Longitudinal examination of youth readmission to mental health inpatient units. Child Adolesc. Ment. Health.

[11] van Alphen N.R., Stewart J.G., Esposito E.C., Pridgen B., Gold J., Auerbach R.P. (2017). Predictors of Rehospitalization for Depressed Adolescents Admitted to Acute Psychiatric Treatment. J. Clin. Psychiatry.

[12] Edgcomb J.B., Sorter M., Lorberg B., Zima B.T. (2020). Psychiatric Readmission of Children and Adolescents: A Systematic Review and Meta-Analysis. Psychiatr. Serv..

[13] von Elm E., Altman D.G., Egger M., Pocock S.J., Gøtzsche P.C., Vandenbroucke J.P., STROBE Initiative (2008). The Strengthening the Reporting of Observational Studies in Epidemiology (STROBE) statement: Guidelines for reporting observational studies. J. Clin. Epidemiol..

[14] Carrà G., Sciarini P., Segagni-Lusignani G., Clerici M., Montomoli C., Kessler R.C. (2011). Do they actually work across borders? Evaluation of two measures of psychological distress as screening instruments in a non Anglo-Saxon country. Eur. Psychiatry.

[15] Carrà G., Bartoli F., Carretta D., Crocamo C., Bozzetti A., Clerici M., Bebbington P.E. (2014). The prevalence of metabolic syndrome in people with severe mental illness: A mediation analysis. Soc. Psychiatry Psychiatr. Epidemiol..

[16] American Psychiatric Association (2013). Diagnostic and Statistical Manual of Mental Disorders.

[17] Bartoli F., Crocamo C., Dakanalis A., Riboldi I., Miotto A., Brosio E., Clerici M., Carrà G. (2017). Association between total serum cholesterol and suicide attempts in subjects with major depressive disorder: Exploring the role of clinical and biochemical confounding factors. Clin. Biochem..

[18] Chowdhury M.Z.I., Turin T.C. (2020). Variable selection strategies and its importance in clinical prediction modelling. Fam. Med. Community Health.

[19] StataCorp (2019). Stata Statistical Software: Release 16.

[20] Azuero A. (2016). A note on the magnitude of hazard ratios. Cancer.

[21] Mounteney J., Griffiths P., Sedefov R., Noor A., Vicente J., Simon R. (2016). The drug situation in Europe: An overview of data available on illicit drugs and new psychoactive substances from European monitoring in 2015. Addiction.

[22] Carrà G., Bartoli F., Brambilla G., Crocamo C., Clerici M. (2015). Comorbid addiction and major mental illness in Europe: A narrative review. Subst. Abus..

[23] Colizzi M., Burnett N., Costa R., De Agostini M., Griffin J., Bhattacharyya S. (2018). Longitudinal assessment of the effect of cannabis use on hospital readmission rates in early psychosis: A 6-year follow-up in an inpatient cohort. Psychiatry Res..

[24] Rezai-Zadeh K.P., Engstrom R.N., Sharma A., Chen Y., Chu J., Cox R.P., Lee M.T. (2019). Generational trends and patterns in readmission within a statewide cohort of clients receiving heroin use disorder treatment in Maryland, 2007–2013. J. Subst. Abus. Treat..

[25] Böckmann V., Lay B., Seifritz E., Kawohl W., Roser P., Habermeyer B. (2019). Patient-Level Predictors of Psychiatric Readmission in Substance Use Disorders. Front. Psychiatry.

[26] Carrà G., Johnson S., Crocamo C., Angermeyer M.C., Brugha T., Azorin J., Toumi M., Bebbington P.E. (2016). Psychosocial functioning, quality of life and clinical correlates of comorbid alcohol and drug dependence syndromes in people with schizophrenia across Europe. Psychiatry Res..

[27] Verdoux H., Liraud F., Gonzales B., Assens F., Abalan F., van Os J. (2001). Predictors and outcome characteristics associated with suicidal behaviour in early psychosis: A two-year follow-up of first-admitted subjects. Acta Psychiatr. Scand..

[28] Bartoli F., Lev-Ran S., Crocamo C., Carrà G. (2018). The interplay between cannabis use and suicidal behaviours: Epidemiological overview, psychopathological and clinical models. J. Psychopathol..

[29] Pompili M., Serafini G., Innamorati M., Biondi M., Siracusano A., Di Giannantonio M., Giupponi G., Amore M., Lester D., Girardi P. (2012). Substance abuse and suicide risk among adolescents. Eur. Arch. Psychiatry Clin. Neurosci..

[30] Zhang X., Wu L.T. (2014). Suicidal ideation and substance use among adolescents and young adults: A bidirectional relation?. Drug Alcohol Depend..

[31] Bartoli F., Crocamo C., Carrà G. (2019). Cannabis use disorder and suicide attempts in bipolar disorder: A meta-analysis. Neurosci. Biobehav. Rev..

[32] Hor K., Taylor M. (2010). Suicide and schizophrenia: A systematic review of rates and risk factors. J. Psychopharmacol..

[33] Carrà G., Scioli R., Monti M., Marinoni A. (2006). Severity Profiles of Substance-Abusing Patients in Italian Community Addiction Facilities: Influence of Psychiatric Concurrent Disorders. Eur. Addict. Res..

[34] Carrà G., Bartoli F., Crocamo C., Brady K.T., Clerici M. (2014). Attempted suicide in people with co-occurring bipolar and substance use disorders: Systematic review and meta-analysis. J. Affect. Disord..

[35] Ostuzzi G., Mazzi M.A., Terlizzi S., Bertolini F., Aguglia A., Bartoli F., Bortolaso P., Callegari C., Caroleo M., Carrà G. (2018). STAR Network Investigators. Factors associated with first- versus second-generation long-acting antipsychotics prescribed under ordinary clinical practice in Italy. PLoS ONE.

[36] MacDonald L., Chapman S., Syrett M., Bowskill R., Horne R. (2016). Improving medication adherence in bipolar disorder: A systematic review and meta-analysis of 30 years of intervention trials. J. Affect. Disord..

[37] Dell’Osso B., Albert U., Carrà G., Pompili M., Nanni M.G., Pasquini M., Poloni N., Raballo A., Sambataro F., Serafini G. (2020). How to improve adherence to antidepressant treatments in patients with major depression: A psychoeducational consensus checklist. Ann. Gen. Psychiatry.

[38] Landolt K., Rössler W., Ajdacic-Gross V., Derks E.M., Libiger J., Kahn R.S., Fleischhacker W.W., EUFEST Study Group (2016). Predictors of discontinuation of antipsychotic medication and subsequent outcomes in the European First Episode Schizophrenia Trial (EUFEST). Schizophr. Res..

[39] WHO Collaborating Centre for Drug Statistic Methodology (2003). Guidelines for ATC Classification and DDD Assignment.

[40] Gavrielov-Yusim N., Friger M. (2014). Use of administrative medical databases in population-based research. J. Epidemiol. Community Health.

[41] Ruggeri M., Lora A., Semisa D., SIEP-DIRECT’S Group (2008). The SIEP-DIRECT’S Project on the discrepancy between routine practice and evidence. An outline of main findings and practical implications for the future of community based mental health services. Epidemiol. Psichiatr. Soc..

[42] Carrà G., Crocamo C., Borrelli P., Tabacchi T.I., Bartoli F., Popa I., Montomoli C., Clerici M. (2017). Area-Level Deprivation and Adverse Consequences in People With Substance Use Disorders: Findings From the Psychiatric and Addictive Dual Disorder in Italy (PADDI) Study. Subst. Use Misuse.

[43] Turecki G., Brent D.A. (2016). Suicide and suicidal behaviour. Lancet.

[44] Carrà G., Crocamo C., Borrelli P., Popa I., Ornaghi A., Montomoli C., Clerici M. (2015). Correlates of dependence and treatment for substance use among people with comorbid severe mental and substance use disorders: Findings from the “Psychiatric and Addictive Dual Disorder in Italy (PADDI)” Study. Compr. Psychiatry.

[45] Calear A.L., Christensen H., Freeman A., Fenton K., Busby Grant J., van Spijker B., Donker T. (2016). A systematic review of psychosocial suicide prevention interventions for youth. Eur. Child Adolesc. Psychiatry.

[46] Singh S.P., Tuomainen H. (2015). Transition from child to adult mental health services: Needs, barriers, experiences and new models of care. World Psychiatry.

[47] Molfenter T., Connor T., Ford J.H., Hyatt J., Zimmerman D. (2016). Reducing psychiatric inpatient readmissions using an organizational change model. WMJ.

